# Roentgenographic Studies of Tissues Involved in Chronic Mouth Infections

**Published:** 1919-01

**Authors:** Arthur D. Black

**Affiliations:** Chicago


### ROENTGENOGRAPHIC STUDIES OF TISSUES INVOLVED IN CHRONIC MOUTH INFECTIONS

BY ARTHUR D. BLACK, A.M., M.D., D.D.S., CHICAGO

In a paper presented before the Section on Stomatology at the New York meeting last year there was included a tabulation covering a critical examination of three thousand radiographic films of the teeth and adjacent bone of the mouths of three hundred adults. During the past year these studies have been continued with results that very closely parallel the previous figures. The present tabulation, which includes that of last year, covers six hundred radiographic mouth examinations, for each of which ten small films were made—a total of six thousand films.

The object of this study is primarily to determine as nearly as possible the average percentage of chronic mouth infections, which are of two types: *First,* those that begin with inflammations of the gingivae and progress along the side of the root toward the apex, destroying the peridental membrane and adjacent alveolar process—chronic suppurative pericementitis; *second,* those which, subsequent to the death of the pulp of the tooth, cause a destruction of the bone about the apex of the root—chronic alveolar abscess. Secondary to the foregoing, other data is being compiled, such as the existing relationship between these mouth infections and the general health, the number of teeth present at various ages, the number of teeth having root fillings, notations as to whether the root fillings are good or poor, and the frequency of alveolar abscess about the apexes of well-filled and poorly-filled roots. These studies are therefore considered to be of fundamental importance from two points of view: *First,* that of chronic mouth infections as a cause of systemic disease, and *second,* that of the dentist's technic in pulp treatment and root filling in relation to the occurrence of alveolar abscess.

An effort has been made to take radiograms of the mouths of persons as we meet them on the street, that is, without previous inquiry as to the condition of their mouths or their health, in order that our figures may represent average conditions. The six hundred persons included in the tabulation consist of about three hundred dental students, about fifty Dental and Medical Reserve Corps officers, about fifty of my own patients, and patients applying for dental service at Northwestern University Dental School. It is believed that the figures represent very close to average conditions for persons of less than forty years of age, but the number of infective foci in persons past forty is doubtless too high, because there are included a few who presented themselves because of systemic conditions that called for mouth examination.

Of the six hundred adults examined, 78 per cent. are shown by the radiograms to have definite areas of bone destruction about the teeth. If we make every possible allowance for variations from the average which these six hundred cases may represent, the fact remains that a high percentage of adults have chronic infections involving the maxillary bones. With our present knowledge of focal infections in relation to systemic disease, these mouth conditions constitute a serious menace to the health-producing power and longevity of the people.

### AREAS OF CHRONIC INFECTION

The accompanying tabulation records areas of chronic infection about the teeth, in 57 per cent. of persons aged from twenty to twenty-four, 64 per cent. aged from twenty-five to twenty-nine, 88 per cent. aged from thirty to thirty-nine, 90 per cent. aged from forty to forty nine and 98 per cent. aged fifty or more. As previously mentioned, the percentages for persons past forty are too high because of the fact that a number of those included in the tabulation presented themselves on account of their general physical condition. Attention is also called to the fact that a considerable number of persons past forty who are edentulous are also free from these infections. None of these were included in this study.

The areas of destroyed bone are given in two groups, under the headings "peridental infections" and "alveolar abscess." The term "peridental infections" is used here to cover all areas of bone destroyed along the sides of the roots of the teeth, from suppurations beginning in the gingivae. The term "alveolar abscess" covers all areas of bone destroyed at the apexes of roots, subsequent to the death of the pulps of the teeth.

The peridental infections are very seldom found in the mouths of persons of less than twenty years. They are to be considered as lesions of adult life only, which are more frequent with advancing years. For the group between the ages of twenty and twenty-four, 13 per cent. had infections of this type; those aged from twenty-five to twenty-nine, 29 per cent.; those aged from thirty to thirtynine, 68 per cent.; those aged from forty to forty-nine, 77 per cent.; those aged fifty or more, 88 per cent. These percentages alone do not tell the full story, as in addition to the increase in the number of persons with advancing years, there is also an increase in the number of areas per person, from four per person of those affected between the ages of twenty and twenty-four to ten per person in those aged fifty or more.

Alveolar abscesses are found in the mouths of persons of all ages. It would be expected that the number would increase with age, except for the fact that the number of abscessed teeth extracted serves to maintain a balance. In the present tabulation for persons aged from twenty to twenty-four, there are recorded 52 per cent. who have abscessed teeth; for those aged from twenty-five to twenty-nine, 51 per cent.; for those from thirty to thirty-nine, 63 per cent.; for those from forty to forty-nine, 59 per cent.; for those of fifty or more, 50 per cent. The number of abscesses per person does not vary much with age, the average being 2.6 per mouth for those aged from twenty to twenty-four having abscesses and the same for those of fifty or more. The lowest was 2.2 abscesses per person for those between the ages of thirty and thirty-nine. It is very interesting to note that last year's tabulation of three hundred persons showed 59 per cent. with alveolar abscess, while for the three hundred added this year only 41 per cent. had alveolar abscess. It is believed that this difference represents in some measure the better realization of the danger of these infections to health, with the result that a larger number of infected teeth have been extracted. No attempt has as yet been made to determine the percentage of abscesses in the mouths of persons of less than twenty years of age.

### RESULTS OF QUESTIONNAIRE

It was not practicable to make thorough physical examinations of these patients, and only a limited number of

* Percentage of abscesses for all root fillings, 45; for good root fillings, 9; for poor root fillings, 63.

reports of such examinations were obtainable. The effort was therefore made to secure by a questionnaire the best possible information as to the physical condition. Inquiry was made as to enlarged finger joints, muscles or joints which were occasionally painful, condition of the nose and throat, inflammation of eyes, attacks of appendicitis, etc. It is appreciated that this information is not very reliable, as it does no more than give a general idea of the health of the persons examined. Of the five hundred and one included in the questionnaire, three hundred and sixty-three are reported as entirely negative as to secondary systemic disorders, seventy-two complained of occasional muscular or joint symptoms, and sixty-six reported well-defined cases of arthritis, nephritis, appendicitis, etc. Forty-two, of the last group of sixty-six, were persons of forty years or older and a considerable number of these presented themselves because of their physical condition. None were hospital cases. The principal value which these figures on systemic conditions give to the tabulation is in showing that the large majority of those examined were in good health, from which fact we may conclude that the number of areas of infection reported are not likely to be far astray in the establishment of average figures, with the exception, already noted, of persons past forty.

The average number of teeth per person is an indication that the mouths probably have had average care by both patient and dentist. It is noted that for the four hundred and eleven persons in the three groups covering ages from twenty to thirty-nine, there is an average of twenty-eight teeth. As the third molars are not constant in development, there is an average loss of less than four teeth per person up to thirty-nine years.

ROOT-CANAL FILLINGS

A feature of this tabulation of especial interest to dentists is the occurrence of alveolar abscess in relation to good and to poor root-canal fillings. For this study the roots of the teeth were divided into two groups: those having large canals and those having small canals. This division was made because of the differences in technic required. The upper central incisor, cuspid, second bicuspid, and lingual roots of molars, the lower cuspid, the first and second bicuspids, and the distal roots of the molars were classed as having large canals. The upper lateral incisor, first bicuspid and buccal roots of molars, and the lower incisors and mesial roots of molars were classed as having small canals.

The radiograms showed sixteen hundred and ninety-five teeth with root fillings. This is 10 per cent. of all of the teeth in the mouths of the persons examined. Of these teeth, three hundred and forty-four were not included in the special study of root fillings, because the shadows were not sufficiently clear for positive reading. Each root of multi-rooted teeth was counted separately. There were recorded a total of three hundred and forty-three good root fillings in large canals, of which thirty-one were abscessed, and five hundred and seventy poor root fillings in large canals, of which three hundred and fifty-six were abscessed. For the small canals, there was one hundred and eighty-four well filled, of which nineteen were abscessed; four hundred and thirteen poorly filled, of which two hundred and seventy-one were abscessed. This gives only 9 per cent. abscessed of all teeth having good root fillings, and 63 per cent. abscessed of all teeth having poor root fillings. Doubtless, some in both groups were abscessed before the root fillings were made. It seems that no better argument could be found to induce dentists to be more painstaking in their root-filling technic, for here is the opportunity to reduce the total of alveolar abscesses to about one-fifth of the present number—an effort very well worth while.

It should be stated that we have been very liberal in classifying these root fillings; if we have erred it has been by placing an excessive number in the "good" columns.

It is appreciated that it is not always possible to determine the condition of a root filling by examination of a roentgenogram. Fillings were classed as good if the root apex was apparently filled or in those cases in which the root filling did not reach to the apex, if no canal was discernible beyond the root filling. By this plan, all questionable fillings were given the benefit of any doubt.

### CHANGES IN DENTAL PRACTICE

The effect of the study of these cases is well shown by the changes that have been noted in the clinic of Northwestern University Dental School. The radiographic service has been increased several fold as compared with a few years ago, a total of more than seventeen thousand films having been made during the past year. In the same period nearly twenty-four thousand teeth were extracted, and the number of artificial crowns and bridges much reduced, while there has been a corresponding increase in the number of artificial dentures made for patients. This is an expression of the changes in dental practice throughout the country. A constantly increasing number of dentists are coming to realize that it is their positive duty to free the mouths of patients from infection, even though this requires the extraction of a number of teeth. But what is of much more importance, there is a determined effort on the part of many to use every possible means of preventing the occurrence of these chronic mouth infections, and those who have seriously undertaken to do so are meeting with a large measure of success. This is preventive dentistry that is worth while, for it is cutting off at the source much systemic disease.

### DISCUSSION

Dr. Eugene S. Talbot, Chicago: Dr. Black speaks of so many cases of root filling and then so many abscesses. I would like to have him tell how he knows these are abscesses. My researches have shown that we must classify, because we know exactly what takes place at the root of the tooth. Many of these cases that are called abscesses are not abscesses but are simply absorptions. In all these experiments that I have been doing the last three or four years, with the microscope, destroying pulps of teeth and then immediately conducting the different drugs that we use through the ends of the roots of the teeth, this sequence invariably takes place. The irritation produces an absorption of the alveolar process. It depends on the amount of the irritation as to where that absorption stops. If it is a slight irritation, the absorption of bone will take place and fibrous tissue will remain around the end of the root of that tooth. That absorption may or may not be very extensive. If the irritation is more severe than what I have just mentioned, the fibrous tissue will be destroyed around a certain area, and that absorption and destruction of fibrous tissue takes on the appearance of what might be mistaken for an abscess under the roentgen-ray. I have noticed that many cases which present conditions that were considered abscesses were not abscesses, but simply absorptions of the alveolar processes and roots and destruction of the peridental membrance at the end of the root of the tooth. If the irritation is still greater, with infection, so that even the fibrous tissue is destroyed, an abscess will form. There are three conditions or stages which we must understand and classify by the roentgen-ray. I have not been able to classify these with the roentgen-ray. We must first examine the case with absorption in order to show the three different conditions that take place. I want Dr. Black to explain these three different conditions, if he can, and why he calls these cases abscesses. The other condition is where the fibrous tissue will remain after the absorption of bone. The next is where the irritation and infection are so great that an abscess will form. *First,* there is absorption of bone and root. The peridental membrane still remains because there is not sufficient irritation to produce a destruction of the peridental membrane and fibrous tissue. *Second,* the peridental membrane and fibrous tissue are destroyed without an abscess forming. *Third,* the fibrous tissue remains around the root of the tooth and an abscess forms in this fibrous tissue and remains after absorption of bone has taken place. These are the three actual conditions that occur and are seen with the microscope.

Dr Thomas L. Gilmer, Chicago: I am in accord with Dr. Talbot's statement that there is in some instances, indication of absorption of the process when no infection exists. After examining radiograms, we are sometimes left in doubt, whether abscesses exist or not. There may be a very limited area of bone destruction followed by a thickening of the pericementum when no infection exists. I am of the opinion, however, that in the majority of these cases the areas are infected. My reason for this belief was strengthened by my study of the bacteriology of alveolar abscess. I extracted a large number of pulpless teeth and secured the *Streptococcus viridans* from almost all cases, when the radiograms showed rarefied areas. Alveolar abscess, when some of the apical peridental membrane has been destroyed, may be cured temporarily by sterilization of the root canal, but it is for a very limited period, because there will be reinfection sooner or later through the organisms being carried to the dead cementum by the blood stream.

Dr. Chalmers J. Lyons, Ann Arbor, Mich.: We must not overlook the mistake that both the dental and medical professions are making today of relying wholly on roentgenograms. I am very much interested in Dr. Talbot's remarks that we should not consider the roentgenographic evidence alone. It is simply one method of making a diagnosis, and I am wondering, if in the report Dr. Black has made, that some distinction might not be made between what he calls an abscess of the roots with poorly-filled canals, and an abscess of a root with well-filled canals. While the roentgenographic evidence would admit of some error, without a complete history of that particular operation, I do not see how he can classify a tooth with a perfectly or well-filled canal having an area of absorption as an abscess. If that is true, if they can be so classified, then our whole root-canal work is in vain.

Dr. Arthur D. Black, Chicago: I endeavored to present certain conditions that were shown by examination of six thousand radiograms, and I believe I qualified that by saying that I presented tabulations of the areas of destruction of bone. Then I stated further that in order to simplify the presentation I called one group peridental destruction, and the other apical destruction. The main point I desire to impress is that over 75 per cent. of the people over twenty have destruction of bone about the roots of teeth.

I would base my judgment in deciding on the treatment of any case showing an area of destruction about the root of the tooth, on the extent of the detachment of the peridental membrane from the surface of the root. If the studies of the histologic function and pathologic changes which take place in these tissues are worth anything to us at all, they certainly tell us that where the peridental membrane has been detached and that detachment has been maintained for a time, there will not be a regrowth of this tissue. If there is a hole in the bone about the end of the root of the tooth, showing that the peridental membrane has been destroyed, then I would not bother to determine whether the area is infected or not, as it would not make a particle of difference in the treatment. If the area is not infected today it may be infected through the circulation tomorrow. I have no doubt that a considerable number of these cases go along for a long time without infection, but they certainly present ideal conditions for reinfection. Granting for the moment that by the use of drugs such an area may be rendered sterile, there could not be more than a very temporary advantage because there is no probability of reattachment of tissues to the root of the tooth. When the radiogram shows an area of bone destroyed immediately about the surface of the root, I believe it should be treated as though it were an abscess whether there is any infection or not.

There can be no question that the number of persons having infections of the bones about the teeth is very much larger that it ought to be. It must be less in the future, and the burden of reducing it is on the dental profession. At the same time, we must remind the members of the medical profession that there are many other regions besides the mouth in which the original foci of infection occur. When we come to realize that 75 per cent. of adults have some infection of the maxillary bones, it seems that three out of every four persons, who go to the physician complaining of some systemic infection, have one or more of these areas in their mouths. I am afraid that too many physicians are jumping at the conclusion that the teeth are the source of the trouble, without making a thorough investigation of other regions, such as the nose and throat, the sinuses of the head, the mastoid, the gall-bladder, the genito-urinary tract, etc.

Every adult who comes to us should have a complete radiographic examination of the mouth. When each dentist takes on himself the responsibility of knowing that the mouths of his patients are free from infection, he will have made real progress in the prevention of systemic disease.—*Dental Summary.*

